# Antenatal screening for chromosomal abnormalities

**DOI:** 10.1007/s00404-022-06477-5

**Published:** 2022-03-13

**Authors:** Karl Oliver Kagan, Jiri Sonek, Peter Kozlowski

**Affiliations:** 1 Department of Obstetrics and Gynaecology, University of Tuebingen, Calwerstrasse 7, 72076 Tübingen, Germany; 2 Department of Obstetrics and Gynecology, Wright State University, Dayton, USA; 3 Praenatal.de—Prenatal Medicine and Genetics, Düsseldorf, Germany

**Keywords:** Cell-free fetal DNA, Ultrasound, First trimester screening, Trisomy

## Abstract

Screening for chromosomal disorders, especially for trisomy 21, has undergone a number of changes in the last 50 years. Today, cell-free DNA analysis (cfDNA) is the gold standard in screening for trisomy 21. Despite the advantages that cfDNA offers in screening for common trisomies, it must be recognized that it does not address many other chromosomal disorders and any of the structural fetal anomalies. In the first trimester, the optimal approach is to combine an ultrasound assessment of the fetus, which includes an NT measurement, with cfDNA testing. If fetal structural defects are detected or if the NT thickness is increased, an amniocentesis or a CVS with at least chromosomal microarray should be offered.

## Introduction

Screening for chromosomal disorders, especially for trisomy 21, has undergone a number of changes in the last 50 years. While screening based on maternal age was the standard in the 1970s and 80 s, it was replaced by biochemical testing such as the triple test in the 1990s. In the 2000s and, also the 2010s, combined first trimester screening (FTS) between 11 + 0 and 13 + 6 weeks’ gestation was used as standard and most effective method of screening. Today, cell-free DNA analysis (cfDNA) is the gold standard in screening for trisomy 21 [1–3]. Increasingly, healthcare systems include cfDNA tests as a part of standard care in pregnancy. Most commonly, the test is performed after FTS. However, some countries, such as the Netherlands use the cfDNA test as ***a*** first-tier screening test [4]. In Germany, the Federal Joint Committee (“Gemeinsamer Bundesausschuss”) decided that each pregnant woman can request the test without a prior risk stratification and regardless of age. At the same time, it is not recommended as a routine screening test.

Regardless of the healthcare system, in prenatal medicine, one must understand the advantages and disadvantages of the different screening tests and the risk profile of each pregnant women. Despite the advantages that cfDNA offers in screening for common trisomies, it must be recognized that it does not adequately address many other chromosomal disorders and any of the structural fetal anomalies.

## Risk for fetal defects and chromosomal disorders

Based on data extracted from the Eurocat registry, the overall prevalence of fetal defects between 2013 and 2019 was 263 per 10,000 births [5]. Out of those, 46.7/10,000 pregnancies had chromosomal defects (live births, stillbirths and terminations), including trisomy 21 (25.1/10,000), trisomy 18 (6.3/10,000), and trisomy 13 (2.3/10,000) [5]. These data illustrate that common chromosomal disorders are important but other fetal defects, including structural defects, are substantially more common.

The individual risk for common autosomal trisomies (21, 18, and 13) increases with maternal age (e.g. the risk for trisomy 21 increases from 1:1250 at 20 years of age to 1:86 at 40). The other chromosomal abnormalities are often referred to as rare chromosomal defects. These include microdeletions and duplications, as well as sex chromosome defects [6]. However, they are not as rare as the name implies. The overall risk for microdeletions and duplications is about 1:270 and it is 1:280 for sex chromosome abnormalities. The prevalence of these chromosomal defects is independent of maternal age. Including the entire spectrum, the risk of any chromosomal disorder in a 20 year old is 1:122. At this age, common trisomies account for only 13.4% of all chromosomal abnormalities. In a 40-year-old woman, the overall risk is 1:40, with the share of trisomies 21, 18, and 13 being 64.5%. This distribution of risks has to be taken into account when counselling women about the most appropriate screening test while keeping in mind that the added benefit of prenatal diagnosis of fetal structural defects can be accomplished only by ultrasound.

## Screening for chromosomal defects

In this section, the strengths and the limitations of the different screening methods for chromosomal abnormalities will be highlighted. We will discuss not only common trisomies, but the entire spectrum of chromosomal disorders. Screening based on maternal age alone will not be addressed as it should not be used as such due to poor test performance [3]. However, maternal age should be included in risk assessment that uses other screening parameters.

### First trimester screening (FTS)

The combined FTS computes individual risks for trisomies 21, 18, and 13 based on maternal age, fetal nuchal translucency (NT) measurement, and maternal serum markers, most commonly free beta-hCG and PAPP-A. Other ultrasound parameters such as the fetal nasal bone, tricuspid valve flow, and blood flow in the ductus venosus can be included in the risk calculation and have been shown to improve the test performance [7–13].

#### Common trisomies

Santorum et al. investigated the test performance of combined FTS in screening for trisomies 21, 18, and 13 in approximately 110,000 pregnancies. For a false-positive rate of 4.6%, the detection rates were 92.1%, 96.4%, and 92.9%, respectively [7]. Median marker levels in euploid and aneuploid pregnancies are listed in Table 1.

**Table 1:** Typical FTS marker profile of euploid and aneuploid fetuses [10]

Karyotype	Median nuchal translucency (mm)	Median-free beta-hCG (MoM)	Median PAPP-A (MoM)
Normal	2.0	1.0	1.0
Trisomy 21	3.4	2.0	0.5
Trisomy 18	5.5	0.2	0.2
Trisomy 13	4.0	0.5	0.3

The combined use of FTS with additional ultrasound markers such as the nasal bone, ductus venosus, and tricuspid valve flow increases the detection rate for trisomy 21 to about 95% and halves the false-positive rate [8, 12–14]. It should be noted, however, that these additional markers represent dichotomous variables that can strongly influence the risk; therefore, they should be used only by those operators who have sufficient expertise to do so [15].

#### Other chromosomal abnormalities

### Increased nuchal translucency

It has been shown that the risk of genetic and structural defects increases with increasing NT measurements [16, 17]. In a recent study, Bardi et al. reported on the outcome of 1901 pregnancies with a fetal NT above the 95th percentile [18]. 43.0% of these fetuses were classified as abnormal. 23.9% had trisomies 21, 18, and 13, and 5.4% had other chromosomal abnormalities detectable by karyotyping alone. Single-gene disorders or abnormalities that can be detected only by microarray analysis were found in 2.0% of the cases each. Structural malformations in the absence of a genetic abnormality were detected in 9.3% of the fetuses. Based on this study, it is predicted that a policy that focuses only on trisomies 21, 18, and 13, such as cfDNA screening, would miss 44.0% of all abnormalities.

These data indicate that the option of a diagnostic test needs to be discussed with patients with a fetus that has an increased NT. However, the cut-off for NT thickness remains controversial. If there are additional defects, there is a broad consensus that an amniocentesis or a CVS should be offered [19]. However, this is less clear for an isolated increased NT. Thresholds of 3.0 mm, 3.5 mm, and the 95th centile have been proposed. Thus far, most study groups have used the 99th percentile, which corresponds to approximately 3.5 mm across the gestational ages when FTS is performed [6, 20, 21]. Regardless of which cut-off is used, the discussion about an appropriate threshold presupposes that the examination is done correctly by trained personnel so that the NT measurement can be accurately compared to the reference range [22]. Table 2 gives an overview of the risk for chromosomal and structural defects stratified according to the NT thickness.

**Table 2:** Risk of chromosomal and structural defects according to the NT thickness (submicroscopic defects = those defects not detectable by routine karyotyping)

NT thickness	Trisomy 21/18/13 (%)	Other chromosomal defects (%)	Submicroscopic defects (%)	Single-gene disorders (%)	Structural defects (%)
95th–3.4	13	1	1	1	6
3.5–4.9	25	3	3	1	11
5.0–6.4	44	13	4	6	11
6.5–7.9	51	9	3	3	17
> 8.0	34	22	1	7	14

The risks are based on the study from Bardi et al. [18]

In a large retrospective study that included more than 81,000 pregnancies, Hui et al. investigated the outcome of fetuses with an NT measurement above 3.0 mm and above the 99th percentile [23]. The rate of atypical chromosomal defects detected only by microarray analysis was 2.1% for the NT range between 3.0 and 3.4 mm and 21.5% for measurements ≥ 3.5 mm. However, in this study, the presence or absence of major structural defects was not taken into account.

Maya et al. performed a study, which was confined to fetuses with an isolated NT enlargement [24]. They compared the proportion of chromosomal defects and pathogenic mutations in three groups: NT of less than 3.0 mm, NT between 3.0 and 3.4 mm and NT of 3.5 mm or more. The prevalence of chromosomal defects was 1.7%, 6.5%, and 13.8%, respectively. Pathogenic mutations that could be detected only by microarray analysis and not by karyotyping or by cfDNA analysis were found in 0.9%, 1.8%, and 2.2% of cases, respectively. As a consequence, the authors recommended that microarray analysis should be performed if the NT thickness is 3.0 mm or more. This is in agreement with the American College of Obstetrics and Gynecology, which makes the same recommendation [6].

Mellis et al. investigated whether exome sequencing (ES) is more appropriate in cases of isolated increase in NT thickness in the first trimester [25]. The study included 213 fetuses with a NT thickness of 3.5 mm or more and where karyotyping and microarray analysis were normal, and subsequently underwent ES. Diagnostic variants (defined in this study as either pathogenic or likely pathogenic) were considered significant. Structural malformations were found in 54 fetuses, of which 22.2% had a diagnostic variant. The remaining cases with apparently isolated increased NT were stratified in three groups. In 37 fetuses, isolated increased NT was initially diagnosed, but structural abnormalities became apparent at a later date. The proportion of fetuses with a diagnostic variant in this group was 32.4%. 111 fetuses with an increased NT did not have structural defect identified even after the first trimester. Two (1.8%) fetuses in this group had an abnormality that could only be detected by exome analysis: one fetus had a uniparental disomy 15 and the other one a mutation in the RERE gene. In seven cases, the pregnancy ended before further screening was carried out. In this group, two (28.6%) fetuses had a pathogenic variant.

In summary, amniocentesis or CVS should be considered if the NT thickness is more than 3.0–3.5 mm. It is clear that diagnostic testing has a high yield in cases where fetal structural defects are identified. However, in recognition of the fact that first trimester fetal anatomic survey is limited by fetal size and that structural abnormalities may not become evident until later, offering diagnostic testing is always a prudent approach to a pregnancy where the nuchal translucency measurement exceeds 3.0–3.5 mm. Microarray analysis is the method of choice as it addresses a larger range of genetic abnormalities than karyotyping alone. However, ES may be useful in those cases where fetal structural anomalies are present, and the first-line testing is negative.

#### Abnormal serum markers

Increased risk for chromosomal abnormalities other than the common trisomies is also seen in cases where the deviation from normal in maternal serum marker levels (free beta-hCG and PAPP-A) is extreme. Therefore, diagnostic testing is recommended if either one of these serum markers falls below 0.2 MoM or if free beta-hCG exceeds 5.0 MoM. In these cases, a microarray analysis should be offered [26].

The basis for this recommendation comes from two Danish studies [27, 28]. Petersen et al. examined retrospectively 193, 638 pregnancies, 1122 of which had an abnormal fetal karyotype. Of those, 262 (23.4%) would have been missed by NIPT alone [28]. The study cohort included 936 and 227 pregnancies where either PAPP-A or free beta-hCG, levels, respectively, were below 0.2 MoM. A fetal chromosomal abnormality was present in 21.4% and 56.6% of the cases, respectively. Out of these chromosomal abnormalities, 23.5% and 37.2% were classified as atypical. In the cohort where free beta-hCG level was ≥ 5.0 MoM, 10.9% of the fetuses were found to be chromosomally abnormal. Of these, 21.1% were labelled as atypical.

Wijngaard et al. also highlighted the importance of the serum markers [29]. This study group included 877 pregnancies examined by microarray analysis and in which the results of the combined FTS was available. The risk for chromosomal abnormalities other than the common trisomies increased by 2.6 and 2.2 times for a free beta-hCG concentration of less than 0.37 MoM or a NT thickness above 3.5 mm, respectively.

### Increased risk after combined FTS

Vogel et al. investigated whether the risk for other chromosomal abnormalities can be assessed based on the FTS risk for common trisomies [30]. In cases where the trisomy 21 risk was between 1:50 and 1:100, 2.7% of abnormal findings were identifiable only by microarray analysis. In a large dataset of more than 100,000 first trimester screening examinations, Lindquist et al. looked for markers for other chromosomal abnormalities [31]. About a quarter of all chromosomal abnormalities were classified as atypical, and the overall prevalence of these abnormalities was 0.1%. The prevalence increased to 4.6% in the group of pregnancies with a combined FTS risk for trisomy 21 of 1:10 or more. The authors also emphasized the importance of the serum markers and highlighted the cut-off of 0.2 MoM.

In summary, a specific FTS risk-based approach in screening for chromosomal abnormalities other than the common trisomies has not been established. However, if the FTS risk is 1:10 or more, it is reasonable to offer diagnostic testing. However, one should keep in mind that the reason for an increase in the FTS risk of this magnitude will be either due to an increase in NT thickness or abnormal serum markers, both of which are considered markers for other chromosomal abnormalities.

### Cell-free DNA analysis

#### Common trisomies

CfDNA screening is currently the best non-invasive method for assessing the risk of trisomies 21, 18, and 13. In a meta-analysis of Gil et al. the detection rates for trisomies 21, 18, and 13 were 99.7%, 97.9%, and 99.0% for a false-positive rate of 0.04% [32]. This has been confirmed by other meta-analyses and a Cochrane review [32–35]. However, it must be stressed that this technology is limited in scope and does not address any of the other fetal problems.

As much as cfDNA technology is convincing in a direct comparison with FTS in screening for the three common autosomal trisomies, it must be pointed out that the methodology also has some weaknesses, such as the test failure rate of about 1–3% [36]. The German Society for Ultrasound in Medicine (DEGUM) has published recommendations for a balanced approach to cfDNA screening. These have been summarized under the “10 golden rules” (Table 3) [19].

**Table 3:** Ten golden rules for the use of cfDNA tests (originally published in German)

According to the legal regulation of most countries, genetic counseling is compulsory prior to and after an NIPT test
NIPT currently allows a reliable risk estimation for trisomies 21, 18, and 13, but not for structural defects. These make up the majority of perinatally relevant anomalies. Most other chromosomal defects and syndromal diseases cannot be detected by NIPT either
NIPT requires an ultrasound examination, ideally before blood sampling and after 12 weeks’ gestation
In case of a fetal defect or increased nuchal translucency, invasive testing (CVS or amniocentesis) is the method of choice to detect chromosomal defects and to avoid unnecessary loss of time until the final diagnosis
The fetal or pregnancy-specific proportion of cell-free DNA in the maternal blood should always be reported. The “fetal fraction” is a quality parameter with a great influence on the test quality
An inconclusive NIPT result needs further clarification. There are more chromosomal defects in this cohort, especially trisomies 13 and 18 and triploidies
NIPT is a screening test. If the NIPT test result is abnormal, a diagnostic test (CVS or amniocentesis) is obligatory. The indication for termination of pregnancy should not be based on NIPT findings only
NIPT for sex chromosomal defects should not be performed routinely
The use of NIPT to determine the risk of rare autosomal aneuploidies, structural chromosomal defects, especially microdeletions and monogenetic diseases in the fetus cannot be generally recommended at present
In twin pregnancies, after assisted reproduction and in obesity, NIPT has a higher failure rate and data on test quality are limited

#### Other chromosomal abnormalities

Over the last few years, attempts have been made to extend the spectrum of cfDNA tests to sex chromosome disorders, rare trisomies, microdeletions/duplications, monogenic disorders, and disorders of chromosome structure. In contrast to combined FTS where other chromosomal defects may be picked up due to extreme marker levels or a significantly increased NT measurement, there are specific cfDNA algorithms, which attempt to identify certain abnormalities other than common trisomies. The fundamental aim of such an approach is to reduce the residual risk of genetic diseases. Maya et al. demonstrated convincingly that extending the spectrum of cfDNA screening does not result in a substantial reduction of residual risk [37]. They examined screening tests for a) trisomies 21,18, and 13, b) addition of sex chromosome disorders, c) addition of common microdeletions/duplications, (including 1p36.3-1p36.2, 4p16.3-4p16.2, 5p15.3-5p15.1, 15q11.2-15q13.1, and microdeletion 22q11.2), and d) genome-wide screening for structural chromosome disorders with more than 7 megabase pairs. In total, 1.2% of the fetuses had chromosomal defects and the applied screening tests resulted in a residual risk of 1.07%, 0.78%, 0.74%, and 0.68%, respectively [38, 39].

In addition, one must keep in mind, that first, adding screening tests for more diseases to the cfDNA panel is likely to increase the overall false-positive rate and second, the prevalence of some of the diseases that can be potentially picked up by an extended cfDNA panel is too low to be included in a reasonable screening program. It should be highlighted that this applies to routine screening in unselected pregnancies. However, cfDNA tests for rare genetic diseases can be useful in the setting where a certain fetal disorder is suspected on ultrasound such as in the case of a suspected FGFR3-related skeletal dysplasia [40].

##### Sex chromosomal disorders

Gil et al. summarized the current performance of tests aimed to detect sex chromosomal defects. Detection and false-positive rates for monosomy X, triple X, XXY, and XYY syndromes were between 95.8 and 100% and 0.004% and 0.41%, respectively [32]. However, these rates should be questioned, since sex chromosomal defects often remain undetected after birth and the population studied was only karyotyped if a chromosomal defect was suspected. Since the study excludes unreported cases, a lower detection rate must be assumed. Lüthgens et al. investigated the confirmation rate of sex chromosomal defects suspected based on cfDNA screening [41]. The overall PPV was 38.9% and was 29.0% for monosomy X, 29.7% for 47, XXX, 57.5% for 47, XXY and 80.0% for 47, XYY. The limited test quality is due to technical and biological limitation such as the inactivation of one maternal X chromosome with increasing maternal age, a higher rate of placental mosaicism, and disorders of the sex chromosomal structure [42].

##### Rare autosomal trisomies

In a large study by Scott et al., which included more than 23,000 cfDNA samples, the prevalence of rare autosomal trisomies (RATs), was 1:835 [43]. Within this group, there were cases with trisomies 7, 16, and 22. Benn et al. reported on RATs in almost 200,000 cfDNA samples [44]. The prevalence was 0.32%, mainly trisomies 7, 15, 16, and 22. In the cases where the outcome was available, 41.1% of the pregnancies resulted in an uneventful live birth and 27.2% in a miscarriage. Fetal structural defects were found in 7.3%. 2.0% had a clinically relevant uniparental disomy and 14.6% of the pregnancies were complicated by fetal growth restriction or low birth weight. The authors advised against the use of the extended cfDNA tests due to the uncertain risks for pregnancy complications and due to the lack of preventive measures as well as monitoring standards.

##### Microdeletions/duplications, especially microdeletion 22q11.2

There are several commercially available screening tests for microdeletions and duplications. However, here we will focus on microdeletion 22q11.2 (DiGeorge syndrome), as this condition is tested for most frequently. In a large retrospective study with more than 80,000 blood samples, the false-positive rate of the SNP-based test was 0.33%. After increasing the sequencing depth, it decreased to 0.07% [45]. In a prospective study by the Tübingen research group, the test failure and false-positive rates of cfDNA testing were noted to be 0.9% and 0.27%, respectively [46]. Schmid et al. evaluated simulated samples (i.e. mixture of plasma of from adults with known 22q11.2 deletion and adult women without this deletion) and actual clinical samples obtained during pregnancy. They reported a detection rate of 75.2% and a specificity of 99.6% [47]. Liang et al. prospectively analyzed more than 94,000 s trimester pregnancies with a genome-wide cfDNA test [48]. In this study, all newborns were examined after birth, and genetic analysis was performed in case of abnormalities. The authors reported on a detection rate of 86.7% and a false-positive rate of 0.001%. However, the prevalence of DiGeorge syndrome was 1:7200, which is significantly lower than expected. This finding casts some doubt on the reported detection rate. In a recent study by Bevilacqua et al. the test performance of the cfDNA test was investigated in 735 pregnancies with a suspected fetal heart defect [49]. In this group, the detection rate was 69.6%, and no false-positive cases were observed.

In addition to the quality of a screening test, the consequence of an abnormal finding must be considered when assessing the usefulness of the test. The structural defects seen in patients with microdeletion 22q11.2 include conotruncal cardiac defects, mild CNS abnormalities, thymic hypoplasia, and polyhydramnios [50]. However, the spectrum of associated defects in mental development is wide and cannot predicted based on the microdeletion alone. This can be challenging for the expectant parents [51].

##### Structural chromosomal defects (“genome wide” screening)

Contrary to its name, the aim of “genome wide” cfDNA screening is not to examine the whole genome. It is designed to detect structural chromosome disorders with about 7 megabase pairs or more. In the Netherlands, the test is being investigated in a prospective population study and has been offered to pregnant women since 2017 (TRIDENT-2 study). The results from 57,000 women were summarized by van der Meij et al. [4]. CfDNA screening indicated a RAT or a structural chromosomal abnormality in 0.18% and 0.16%, respectively. The positive predictive value of the abnormal cfDNA test result for a fetal chromosomal defect was 6% and 32%, respectively. The quasi-implementation of “genome wide” screening into the care of all pregnant women in the Netherlands has been critically questioned due to the many unanswered questions regarding the further management of pregnancies and counselling of the couple [52].

### Useful combinations of FTS and cfDNA analysis

As shown above, both FTS and cfDNA analysis play important and additive roles in screening for chromosomal disorders. Therefore, several study groups have assessed the combined use of the two techniques. Usually, the screening algorithm starts with FTS followed by cfDNA testing in a subgroup, which is selected based on the FTS results. Miltoft et al. investigated such a two-stage approach. Combined FTS was performed in all pregnant women and cfDNA screening for trisomies 21, 18, and 13 was performed if the FTS risk fell between 1:100 and 1:1,000 [53]. If the risk was less than 1:1000, no further tests were offered; if the risk was greater than 1:100, or if the cfDNA analysis was abnormal, a diagnostic test was performed. This model was compared with the use of combined FTS alone, at a single threshold of 1:300. All pregnancies affected by trisomy 21 were detected by both screening policies. However, the false-positive rate of the two-stage model was 1.2% while it was 3.0% with the classical approach. Gil et al. offered cfDNA screening to women with an intermediate FTS risk between 1:101 and 1:2,500 [54]. Women in the high-risk group (risk of ≥ 1:100), were asked to choose between a diagnostic and a cfDNA test. The approach resulted in a real detection rate of 91.5% for trisomy 21, with only 38% of women with a risk above 1:100 opting for a diagnostic test. In the intermediate risk group (1:101–1:2,500), 91.5% of pregnant women opted for cfDNA testing. Overall, an amniocentesis or a CVS was performed in 2.7% of cases.

In a study from 2015, Kagan et al. used prospectively collected FTS results from nearly 87,000 pregnancies with a risk calculation based on fetal NT and ductus venosus flow [55]. There were 324 fetuses with trisomy 21. The assumption was made that cfDNA testing would be used in women at risk of 1:100 to 1:2,500, and that the detection and false-positive rates of cfDNA screening would be 99.0% and 0.08%, respectively. Using such an approach, the detection and false-positive rates were calculated to be 96.0% and 2.3%, respectively. If the upper risk threshold is raised to 1:10, the detection rate remains almost unchanged, but the false-positive rate drops to 0.8%. In the studies mentioned above, the proportion of women in the intermediate risk group who would be offered cfDNA screening range from 11.4 to 29.9%.

In a prospective study, the Tübingen research group investigated the test performance of cfDNA screening for all pregnancies after a detailed ultrasound examination in the first trimester [20, 21]. In cases of increased NT or fetal defects, a diagnostic test instead of cfDNA screening was carried out. The aim of our study was to compare the false-positive and invasive testing rate of such a policy with classical combined FTS. In 2.0% of the cases, the ultrasound examination was abnormal. In the group where the first trimester ultrasound did not reveal any structural abnormalities, cfDNA screening resulted in a false-positive and invasive testing rate of 0% and 0.3%, respectively. In the FTS group, the rates were 2.5% and 1.7%, respectively.

### Role of the early anomaly scan at 11–13 weeks’ gestation in screening for chromosomal abnormalities

Several studies have addressed the test performance of an early anomaly scan in screening and diagnosis of fetal structural defects [56]. In a meta-analysis by Karim et al. the authors showed that in a low-risk population the detection rate for all anomalies was 32.4% and was 46.1% for severe malformations [57]. The overall detection rate in a high-risk cohort was 61.2%. The detection rate for cardiac defects was 55.8% [58]. In a large study by Syngelaki et al. which was not included in the above meta-analysis, the overall detection rate of fetal structural anomalies in the first trimester was 27.6% [59]. In a recent single center study, which included more than 50,000 low-risk pregnancies, the overall detection rate was 43%. The authors emphasized the importance of a rigid examination protocol that specifies the structures to be examined [60].

The benefits of assessing the fetal sonomorphology even before a cfDNA test was summarized in a recent editorial [61]. The following are the main points regarding the first trimester ultrasound evaluation:

- Common trisomies constitute a relatively small proportion of the spectrum of fetal defects. Ultrasound plays an important role in screening and diagnosis of other chromosomal defects, genetic and non-genetic syndromes, and fetal structural defects.
- Early anomaly scan may increase the overall detection of fetal defects in pregnancy.
- In some cases, especially in obese women, an early anomaly scan performed transvaginally can provide better visualization than the standard second trimester examination.
- In cases of cfDNA test failure, FTS as part of the early anomaly scan can provide a reliable risk assessment for trisomy 21.
- Extended genetic analysis such as exome sequencing takes time and may be initiated based on the results of an anatomic scan at 11–13 weeks. Consequently, the patient still had the full range of reproductive options available when the results are known.
- If a fetal problem is found on a comprehensive evaluation early in pregnancy, termination can also be done early in pregnancy. This is a safer procedure and tends to be less traumatic for the patient.
- An early fetal anatomic ultrasound, including NT measurement, is an essential component of a comprehensive fetal evaluation, which provides the patient reliable information regarding the fetus at an earliest possible time in pregnancy. It is self-evident that this is what most patients desire.

## Fetal defects and associated genetic syndromes

The effectiveness of an early anomaly scan in screening for trisomies 18 and 13, triploidy and monosomy X was demonstrated by Wagner et al. [62]. At least one malformation was found in 1.3% of the euploid and in 83.5% of the aneuploid fetuses. Using the combination of ultrasound markers and anomalies seen on an early ultrasound, a detection rate for these chromosomal defects was 95.6% and the false-positive rate was 3%. Thus, the detection was on the level of cfDNA analysis with the advantage of also detecting other non-chromosomal disorders. However, the false-positive rate in screening for these chromosomal defects was higher than for cfDNA testing.

Miranda et al. studied 226 pregnancies with an NT thickness of 3.5 mm or more. In this group, 84 fetuses were found to have genetic abnormalities [63]. A cfDNA analysis would have identified the reason for the increased NT in 81.0% of the cases. Conversely, the genetic abnormalities in the remaining 19.0% would not have been detected if cfDNA testing would have been employed without the prior ultrasound evaluation. Most of the fetuses in this group had abnormalities that are recognized as indications for a CVS or an amniocentesis. Of the 142 fetuses with an increased NT but without a genetic disorder, 23.9% had structural malformations. Syngelaki et al. focused on the risk of atypical chromosomal abnormalities in cases with major anomalies. Of the fetuses with holoprosencephaly, omphalocele, or megacystis that had a chromosomal abnormality, the chromosomal abnormality was not detectable by cfDNA analysis in 20.6%, 9.4%, and 20%, respectively. The authors did not use exome analysis, which may have further increased the proportion of atypical chromosomal abnormalities [64].

Table 4 summarizes selected screening policies in screening for trisomy 21 and highlights the detection rates for other chromosomal abnormalities as well as the advantage***s*** and disadvantage***s*** of each screening policy.

**Table 4:** Selected policies in screening for trisomy 21 and other chromosomal abnormalities

First trimester screening policy	Description	DR/FPR (%) trisomy 21	Other chromosomal abnormalities	Advantage	Disadvantage
Combined screening	MA + GA, fetal NT, free beta-hCG & PAPP-A in all patients	92/4.6 [7]	DR > 93% for trisomies 18/13, monosomy X, triploidy: DR > 50%* for other aneuploidies	Effectiveness proven in multiple studies, easy to use, detects multiple pregnancies and chorionicity, abnormal markers can detect other chromosomal and non-chromosomal abnormalities, establishes accurate GA	Lower test performance than cfDNA, requires training
Contingent screening with new markers	Combined screening with *NB,* DV or TR in all women or in the intermediate risk group *only*	93–96/2.5 [8, 12, 13]	DR > 92% for trisomies 18/13, monosomy X and triploidy	See combined screening, improves detection of cardiac defects	Some variables are dichotomous and have a high likelihood ratio, requires additional training
Combined screening with new markers and anomaly scan	Combined screening with new markers and detailed anomaly scan in all patients	96/5% (without serum biochemistry) [14, 62]	DR 96% for trisomies 18/13, monosomy X, triploidy (without biochemistry)	See contingent screening with new markers, detection of other chromosomal abnormalities, detects about 50% of serious fetal structural defects	See contingent screening with new markers, requires additional training and skill in first trimester ultrasound evaluation
Cell-free DNA only	Cell-free DNA screening for all without prior ultrasound in all patients	99/0.1 [32]	DR > 96% for trisomies 18/13, monosomy X; possible test extension to other chromosomal abnormalities	High-test performance for common trisomies, easy to use	Standard test does not include chromosomal abnormalities other than common trisomies, relatively high test failure rate, does not provide any other information regarding the pregnancy
Cell-free DNA and anomaly scan with NT	Anomaly scan and NT assessment prior to cfDNA screening in all patients	> 99/0.1 [20, 21]	> 96%	Combines the advantages of cfDNA screening with the ones from combined screening with anomaly scan, offers reflex testing for cfDNA test failure	Requires training and skill in first trimester ultrasound evaluation
Contingent combined screening with cfNDA	Combined screening in all patients with cfDNA in the intermediate risk group	100/1.2 [53]	100 ´	Combines the advantages of contingent screening with the ones from cfDNA screening Restricts CfDNA screening to intermediate risk group	Requires training

*MA* maternal age, *GA* gestational age, *DV* Ductus venous flow, *TR* tricuspid flow, *NB* nasal bone assessment

## Summary

It is clear that a comprehensive first trimester fetal evaluation is much more than screening for trisomy 21, trisomy 18, and trisomy 13. Using cfDNA exclusively results in the failure to diagnose the majority of fetal problems. In the first trimester, the optimal approach is to combine an ultrasound assessment of the fetus, which includes an NT measurement, with cfDNA testing. The inclusion of maternal serum biochemical markers likely provides added benefit. If fetal structural defects are detected or if the NT thickness is increased, an amniocentesis or a CVS with at least chromosomal microarray should be offered.

## Data Availability

Not applicable as this is a review paper.
